# Supplementary material for: MetaRibo-Seq measures translation in microbiomes
Source: Nat Commun. 2020 Jun 29;11:3268. doi: 10.1038/s41467-020-17081-z (PMC7324362; doi:10.1038/s41467-020-17081-z)
Supplement: Supplementary file 10 — Supplementary Data 7 [file 41467_2020_17081_MOESM10_ESM.zip › File2/Confidence_High_Taxonomy/387752_out.krona.html]

Javascript must be enabled to view this page.

members
magnitude
magnitudeUnassigned
count
unassigned
taxon
rank

387752\_out

171

2
171
superkingdom

170
1239
phylum

class
186801
170

186802
170
order

170
541000
family

170
216851
genus


SRS014459\_contig\_number\_30363SRS023526\_contig\_number\_37788SRS075773\_contig\_number\_26558SRS104084\_contig\_number\_12857SRS893172\_contig\_number\_11459
5
1971605
species

species
1897006
6

SRS016954\_contig\_number\_22565SRS017521\_contig\_number\_18488SRS019445\_contig\_number\_12103SRS049402\_contig\_number\_2408SRS054956\_contig\_number\_10239SRS147919\_contig\_number\_11124

853
157
species

SRS011239\_contig\_number\_27196SRS012969\_contig\_number\_15954SRS013687\_contig\_number\_contig-100\_3339.174920SRS013940\_contig\_number\_17620SRS013951\_contig\_number\_contig-100\_2998.51357SRS013965\_contig\_number\_18761SRS014235\_contig\_number\_contig-100\_1568.263148SRS014287\_contig\_number\_contig-100\_2964.108616SRS014412\_contig\_number\_contig-100\_1271.119963SRS014459\_contig\_number\_1346SRS014683\_contig\_number\_contig-100\_362.119990SRS014855\_contig\_number\_1666SRS014855\_contig\_number\_1837SRS014923\_contig\_number\_37651SRS014948\_contig\_number\_22468SRS015065\_contig\_number\_182SRS015264\_contig\_number\_14248SRS015431\_contig\_number\_contig-100\_5458.424676SRS015579\_contig\_number\_contig-100\_456.154967SRS015663\_contig\_number\_72SRS017307\_contig\_number\_18506SRS017433\_contig\_number\_contig-100\_3602.104323SRS018351\_contig\_number\_1021SRS018623\_contig\_number\_863SRS018836\_contig\_number\_1957SRS018836\_contig\_number\_25282SRS018984\_contig\_number\_23312SRS019030\_contig\_number\_18396SRS019286\_contig\_number\_6058SRS019693\_contig\_number\_2793SRS020394\_contig\_number\_17331SRS020869\_contig\_number\_contig-100\_1332.222332SRS021484\_contig\_number\_20775SRS022071\_contig\_number\_3956SRS022524\_contig\_number\_15609SRS022609\_contig\_number\_contig-100\_862.269945SRS023526\_contig\_number\_38651SRS023914\_contig\_number\_28573SRS024492\_contig\_number\_contig-100\_2675.117304SRS043411\_contig\_number\_13629SRS045244\_contig\_number\_8700SRS045645\_contig\_number\_contig-100\_1212.175247SRS046068\_contig\_number\_4314SRS046369\_contig\_number\_11478SRS046502\_contig\_number\_3084SRS047014\_contig\_number\_340SRS047433\_contig\_number\_24063SRS048164\_contig\_number\_22793SRS048870\_contig\_number\_contig-100\_7947.104029SRS049446\_contig\_number\_15212SRS049773\_contig\_number\_24471SRS049959\_contig\_number\_contig-100\_2467.249241SRS049995\_contig\_number\_38791SRS050422\_contig\_number\_32976SRS050752\_contig\_number\_28395SRS050925\_contig\_number\_contig-100\_1001.193802SRS050998\_contig\_number\_17335SRS052697\_contig\_number\_45350SRS055982\_contig\_number\_19570SRS056273\_contig\_number\_contig-100\_3191.186027SRS058723\_contig\_number\_contig-100\_3595.37363SRS062654\_contig\_number\_21185SRS062701\_contig\_number\_3535SRS063370\_contig\_number\_contig-100\_4241.73311SRS063518\_contig\_number\_5244SRS063985\_contig\_number\_11827SRS064276\_contig\_number\_contig-100\_2278.214298SRS065176\_contig\_number\_contig-100\_1887.71047SRS074964\_contig\_number\_3506SRS075078\_contig\_number\_8675SRS075773\_contig\_number\_contig-100\_3663.84924SRS075878\_contig\_number\_7589SRS076756\_contig\_number\_15095SRS076976\_contig\_number\_7657SRS077194\_contig\_number\_11575SRS077231\_contig\_number\_10633SRS077294\_contig\_number\_contig-100\_2397.126282SRS077454\_contig\_number\_contig-100\_4521.77954SRS077552\_contig\_number\_26560SRS077849\_contig\_number\_31075SRS078176\_contig\_number\_contig-100\_929.143454SRS097889\_contig\_number\_contig-100\_1471.175057SRS097920\_contig\_number\_3034SRS097920\_contig\_number\_3946SRS097958\_contig\_number\_21267SRS098571\_contig\_number\_37424SRS098655\_contig\_number\_14042SRS098717\_contig\_number\_22778SRS098881\_contig\_number\_19458SRS100021\_contig\_number\_contig-100\_807.86376SRS101376\_contig\_number\_contig-100\_121.135055SRS101433\_contig\_number\_15342SRS1041038\_contig\_number\_16607SRS1041136\_contig\_number\_12351SRS1041136\_contig\_number\_12971SRS1041145\_contig\_number\_38926SRS104311\_contig\_number\_13212SRS104400\_contig\_number\_24044SRS104636\_contig\_number\_31243SRS104975\_contig\_number\_16825SRS105082\_contig\_number\_10758SRS105082\_contig\_number\_20140SRS105153\_contig\_number\_13137SRS1055043\_contig\_number\_26763SRS1055049\_contig\_number\_17075SRS1055076\_contig\_number\_16528SRS142503\_contig\_number\_contig-100\_5061.279038SRS142712\_contig\_number\_2424SRS142890\_contig\_number\_1805SRS142980\_contig\_number\_452SRS143148\_contig\_number\_16880SRS143342\_contig\_number\_21182SRS143417\_contig\_number\_25901SRS143466\_contig\_number\_15995SRS143523\_contig\_number\_16066SRS143523\_contig\_number\_26468SRS143780\_contig\_number\_24994SRS143876\_contig\_number\_3445SRS143876\_contig\_number\_17172SRS143895\_contig\_number\_1192SRS143895\_contig\_number\_22150SRS144135\_contig\_number\_468SRS144135\_contig\_number\_16141SRS144183\_contig\_number\_4216SRS144297\_contig\_number\_contig-100\_1722.108593SRS144506\_contig\_number\_27SRS144506\_contig\_number\_25111SRS144537\_contig\_number\_4516SRS144537\_contig\_number\_17989SRS144714\_contig\_number\_12533SRS144714\_contig\_number\_contig-100\_10671.92330SRS144753\_contig\_number\_contig-100\_4416.50125SRS146764\_contig\_number\_contig-100\_1836.187169SRS146812\_contig\_number\_36776SRS146888\_contig\_number\_14998SRS147139\_contig\_number\_26721SRS147766\_contig\_number\_2161SRS147919\_contig\_number\_557SRS147977\_contig\_number\_5334SRS148091\_contig\_number\_contig-100\_925.97691SRS148159\_contig\_number\_30145SRS148196\_contig\_number\_41001SRS148721\_contig\_number\_20826SRS148784\_contig\_number\_contig-100\_3362.148986SRS148874\_contig\_number\_4883SRS149075\_contig\_number\_682SRS149075\_contig\_number\_15746SRS149879\_contig\_number\_5346SRS893231\_contig\_number\_contig-100\_3184.3185SRS893252\_contig\_number\_17891SRS893259\_contig\_number\_contig-100\_618.54449SRS893327\_contig\_number\_10927SRS893342\_contig\_number\_10324SRS893358\_contig\_number\_14057SRS893378\_contig\_number\_11063SRS893378\_contig\_number\_18015SRS971275\_contig\_number\_3880


SRS077194\_contig\_number\_2304SRS140492\_contig\_number\_13883
1262898
2
species

phylum
976
1

1
200643
class

order
171549
1

1
2005525
family

genus
375288
1

species
46503
1

SRS018936\_contig\_number\_11416
